# Malnutrition and Sarcopenia in Patients with Neuroendocrine Tumors: A Comprehensive Review of Evidence

**DOI:** 10.3390/biom15121746

**Published:** 2025-12-17

**Authors:** Kalliopi Anna Poulia, Ariadni Spyroglou, Odysseas Violetis, George Mastorakos, Krystallenia I. Alexandraki, Athanasios G. Papavassiliou

**Affiliations:** 1 Laboratory of Dietetics and Quality of Life, Department of Food Science and Human Nutrition, Agricultural University of Athens, 11855 Athens, Greece; lpoulia@aua.gr; 2 Second Department of Surgery, ‘Aretaieion’ Hospital, Medical School, National and Kapodistrian University of Athens, 11528 Athens, Greece; aspyroglou@aretaieio.uoa.gr (A.S.); odysseas@med.uoa.gr (O.V.); gmastorak@med.uoa.gr (G.M.); 3 Unit of Endocrinology, Diabetes Mellitus and Metabolism, ‘Aretaieion’ Hospital, Medical School, National and Kapodistrian University of Athens, 11528 Athens, Greece; 4 Department of Biological Chemistry, Medical School, National and Kapodistrian University of Athens, 11527 Athens, Greece; papavas@med.uoa.gr

**Keywords:** neuroendocrine neoplasms, sarcopenia, malnutrition, nutrition interventions, muscle mass

## Abstract

Neuroendocrine neoplasms (NENs) are rare and heterogeneous tumors with heterogeneity in morphology and molecular profile and consequently resulting in a heterogeneous biological behavior. They have a more indolent natural history compared to the classic cancer and may emerge in any site of the human body, but usually they have gastroenteropancreatic (GEP) or bronchopulmonary (BP) origin. When NENs are well differentiated, they are called neuroendocrine tumors (NETs) as opposed to poorly differentiated neuroendocrine carcinomas (NECs). They may secrete a bioactive molecule resulting in a secretory syndrome or they may not be associated with any secretory product, defining functional and non-functional NENs. The hormonal hypersecretion syndromes, the chronic symptom burden, the tumor-related inflammation, and the treatment side effects impair nutritional intake and absorption while increasing metabolic needs. The present comprehensive narrative review is summarizing established and emerging methods of nutritional and body composition assessment, and the recent evidence of interventions for sarcopenia and malnutrition in patients with NETs. Early identification and management of malnutrition and sarcopenia are fundamental steps to improve quality of life and clinical outcomes in these patients during the long natural history of these neoplasms.

## 1. Introduction

### 1.1. Background

Neuroendocrine neoplasms (NENs) are rare and heterogeneous tumors with diverse profiles in terms of morphology and molecular profile, resulting in different biological phenotypes. NENs are arising from neuroendocrine cells throughout the body, but their main origin is gastroenteropancreatic (GEP) and bronchopulmonary (BP) [1]. When NENs are well differentiated, they are called neuroendocrine tumors (NETs) as opposed to poorly differentiated neuroendocrine carcinomas (NECs). They may secrete a bioactive molecule resulting in a secretory syndrome, or they may not be associated with any secretory product, defining functional and non-functional NENs [2].

Their incidence and prevalence have increased over recent decades worldwide. Improvements in diagnostic imaging, endoscopy, histopathology, and overall disease awareness have contributed to early and more frequent detection [3,4,5]. They have a slow progression along with a more indolent natural history compared to classic cancer, but they present a metastatic potential in up to 20% of patients. As survival has improved because of earlier diagnosis and expanding systemic and locoregional treatment options, NETs are considered chronic oncologic conditions, resulting in a shift in clinical priorities toward the maintenance of health-related quality of life (QoL), functionality, and the preservation of muscle mass, the prevention of nutrition-related problems and malnutrition [6,7,8].

Within this context, deterioration of nutritional status and body composition—including malnutrition, cancer cachexia, and sarcopenia—is of particular concern in populations with NET. As the overall survival of these patients has increased, they are sustaining the cumulative effects of treatment exposures and persistent hormonal or tumor-related symptoms, which have a negative impact on physical and nutritional status [9].

### 1.2. Malnutrition, Cachexia, and Sarcopenia in NETs

Malnutrition generally refers to insufficient nutrient intake and absorption and, as a result, suboptimum coverage of nutritional requirements, leading to measurable changes in body mass, body composition, and physical or mental function [10]. On the other hand, cancer cachexia describes a multifactorial syndrome driven by systemic inflammation, metabolic dysregulation, anorexia, and reduced food intake, resulting in significant loss of muscle mass (with or without loss of fat mass) that cannot be fully reversed by conventional nutritional support, especially in later stages [11]. Sarcopenia, originally defined in geriatrics, denotes a progressive loss of muscle mass and strength associated with adverse outcomes [12,13]. In oncology is now frequently assessed by using imaging-based techniques evaluating muscle quantity and quality, combined with measures of muscle function or performance. Across solid tumors, sarcopenia, cachexia, and broader forms of disease-related malnutrition are consistently associated with increased treatment toxicity, impaired physical function, poorer quality of life, and reduced survival, underscoring the need to understand their determinants and management, specifically in NETs [14].

NETs increase the risk of nutrition-related complications. Hormone hypersecretion syndromes—such as carcinoid syndrome, gastrinoma, insulinoma, glucagonoma, or VIPoma—can induce chronic diarrhea, steatorrhea, vomiting, hypoglycemia, or increased catabolic activity, directly reducing nutrient absorption and increasing energy and protein requirements [15]. Disease chronicity, often spanning years to decades, exposes patients to medical and surgical procedures (including bowel or pancreatic resections), somatostatin analogs (SSAs), peptide receptor radionuclide therapy (PRRT), targeted agents, and cytotoxic chemotherapy, all of which may have a negative impact on appetite, digestion, exocrine pancreatic function, bile acid physiology, and intestinal motility [16]. As a result, many patients with NET experience a cumulative nutritional burden characterized by involutional weight loss, micronutrient deficiencies, altered body composition with muscle depletion, and fluid imbalance, often masking weight loss when traditional anthropometric indices such as body mass index (BMI) are used in isolation [17]. Although nutritional support in oncology is well established, the application of Global Leadership Initiative on Malnutrition (GLIM) malnutrition and European Working Group on Sarcopenia in Older People 2 (EWGSOP)2 sarcopenia diagnostic criteria, published data on NET-specific populations remain limited. Therefore, this review can be the basis of a more comprehensive evaluation of the applicability of these consensuses on NET specific populations. Nutrition-related dysregulations have important implications for patient-centered outcomes in NETs. Loss of skeletal muscle mass and strength contributes to declines in physical function, fatigue, and limited QoL [16,18]. Moreover, they contribute to reduced capacity to tolerate repeated cycles of systemic therapy or major surgery, potentially limiting access to optimal oncologic treatment sequences [19]. Malnutrition and sarcopenia have been linked in broader oncologic cohorts to increased postoperative complications, dose-limiting toxicities, longer hospital stays, and diminished health-related QoL, patterns that are highly relevant to patients with NETs given their prolonged survivorship and frequent multimodal therapy [20,21]. Furthermore, variable symptom burdens—such as diarrhea, abdominal pain, and early satiety—can result in inadequate oral intake, reinforcing nutritional decline, functional impairment, and psychological distress.

The present narrative review aims to synthesize current evidence on interventions targeting malnutrition and sarcopenia in adults with NETs. In the context of this review, malnutrition is defined in line with contemporary consensus frameworks as a state of inadequate nutritional intake and/or assimilation leading to altered body composition, diminished physical function, and adverse clinical outcomes, and, where available, was defined using the GLIM criteria [10] or validated oncology nutrition tools such as Nutritional Risk Screening 2002 (NRS-2002), Malnutrition Universal Screening Tool (MUST), or Patient-Generated Subjective Global Assessment (PG-SGA) [21], often in combination with clinically relevant weight loss and BMI thresholds. Sarcopenia is considered a muscle-centered phenotype characterized by low skeletal muscle mass in conjunction with reduced muscle strength and/or physical performance; in the included NET literature it was most commonly identified using EWGSOP/EWGSOP2 criteria [13] or by cross-sectional imaging (typically CT-derived skeletal muscle indices) at predefined vertebral levels, acknowledging that some studies relied primarily on quantitative imaging cut-offs without comprehensive functional assessment. Emerging interventions are defined as novel or not yet widely implemented strategies specifically targeting malnutrition, sarcopenia, or the underlying metabolic and inflammatory milieu in NETs, including but not limited to microbiome-modulating approaches, ghrelin receptor agonists, myostatin-pathway inhibitors, and digital or artificial-intelligence-supported tools designed to personalize nutritional and physical activity management beyond standard dietetic counseling and routine oncologic care. By integrating current evidence and outlining pragmatic strategies for clinical practice, this review seeks to support the development of individualized, multidisciplinary approaches to prevent, detect, and treat malnutrition and sarcopenia in patients with NETs, with the goal of enhancing QoL, treatment tolerance, and long-term survivorship.

## 2. Pathophysiology of Malnutrition and Sarcopenia in NETs

### 2.1. Tumor-Related Mechanisms

**Figure 1:**
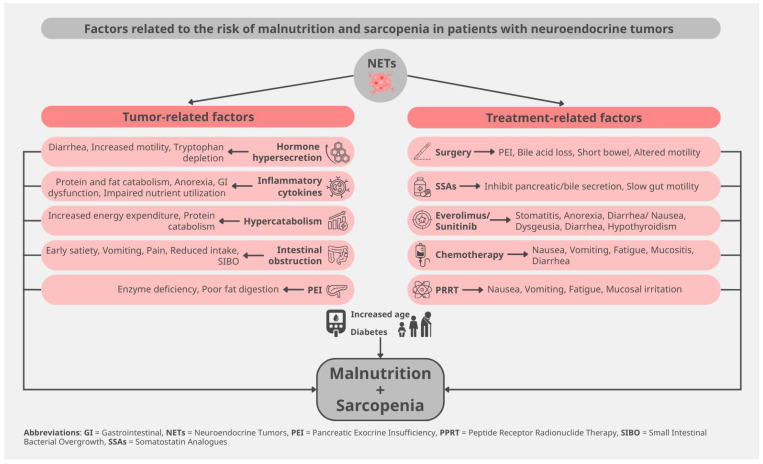
Risk factors for malnutrition and sarcopenia in patients with neuroendocrine tumors.

Patients with NETs increasingly present malnutrition, given the improved overall survival of these neoplasms. Thus, 5–38% of patients present with weight loss, whereas 5–12% display low BMI [9,19]. Sarcopenia is particularly frequent in patients with GEP-NET, reaching 61–87% of all affected patients [22,23]. This extraordinarily high prevalence of sarcopenia can be explained not only by classic tumor-induced catabolic effects but also due to the secretory function of these neoplasms (Figure 1).

When serotonin and othe bioactive substances (histamine, bradykinin, kallikrein) are secreted by NET cells, are causing carcinoid syndrome with diarrhea or bronchospasm, flushing, and carcinoid heart disease. Each of these symptoms can lead to malnutrition. Muscle wasting is now being acknowledged as one of the metabolic consequences of carcinoid syndrome due to uncontrolled diarrhea, which also causes electrolyte loss, dehydration, fatigue, and thereby also reduced appetite [24]. Bronchospasm leads to fatigue and difficulty eating during dyspnea episodes, but also to increased energy expenditure. Similarly, carcinoid heart disease also causes anorexia due to fatigue but also due to right cardiac failure, with hepatic congestion and ascites [16]. Flushing, also dependent on specific food consumption, leads to food avoidance and thereby to reduced appetite [25]. As the amino acid tryptophan is the main precursor for serotonin production in carcinoid syndrome, the synthesis of the water-soluble vitamin B3 (niacin) from the same precursor is impaired in these patients, leading to niacin deficiency, with dermatitis, chronic diarrhea, and dementia [25,26,27].

Gastrin hypersecretion in Zollinger-Ellison syndrome causes peptic ulcers and steatorrhea, resulting in impaired nutrient uptake, whereas watery diarrhea syndrome related to VIPoma causes, among others, hypokalemia, with muscle weakness, metabolic acidosis, and hypochlorhydria, thus, mineral loss, leading to bone demineralization [16,17,18,19,20,21,22,23,24,25,26,27,28]. On the other hand, glucagonomas, besides diarrhea, cause zinc deficiency with glossitis, stomatitis, impaired food intake, diabetes with catabolic stress, increased hepatic gluconeogenesis, lipolysis, and muscle wasting, thus leading to cachexia [15,29]. In line with these observations, somatostatinomas present with diabetes, diarrhea, and hypochloridria, resulting in muscle wasting and sarcopenia. Finally, unlike all previous functioning NETs, insulinoma-related malnutrition is not linked to weight loss. In fact, the inappropriate weight gain upon hypoglycemia symptoms suggests a metabolic impairment, with increased fat mass [30]. Taken together, all these hormones affect overall nutritional status in NETs, contributing to malabsorption. In addition, the released hormones and cytokines exert direct effects on muscle turnover, leading to muscle wasting, altogether attenuating sarcopenia.

It has been demonstrated that the expression levels of ghrelin-O-acyltransferase, the activating enzyme of ghrelin, were significantly higher in patients with NET presenting with weight loss, whereas the ghrelin levels and the two splice variants of its receptor [growth hormone secretagogue receptor 1-a (GHSR1a) and growth hormone secretagogue receptor 1-b (GHSR1b)] also present altered expression in NETs. Ghrelin is an orexigenic gut hormone that antagonizes muscle catabolism occurring in cancer cachexia but also has a role in processes related to tumor proliferation and progression [23].

Still, not only functioning NETs but also hormonally inactive tumors are associated with malnutrition. The presence of malignancy is associated with nonspecific symptoms such as fatigue, low appetite, nausea, and weight loss. In parallel, tumor inflammation, acknowledged as one of the contributing factors in cachexia, also exerts its effects in patients with NETs. Inflammation can either promote or suppress neuroendocrine carcinogenesis, with contrasting results in tumor progression vs. nutritional status [17]. It has been observed that patients with NEC suffer much more frequently from malnutrition in comparison to patients with NETs, possibly in the context of a hypermetabolic state [19,22,31]. Furthermore, depending on the tumor localization, mass effects can affect food intake and/or absorption. In this context, gastric NETs cause nausea, vomiting, early satiety, pain, and/or obstruction, whereas pancreatic NETs can cause altered glucose metabolism, impaired pancreatic exocrine function, biliary obstruction, and thereby malabsorption [27]. In the case of small intestine neoplasms tumor mass can cause bowel obstruction and diarrhea, and, in particular, the mesenteric metastases can lead to desmoplastic reaction with fibrosis and ischemia, causing postprandial abdominal pain, malabsorption, and sarcopenia [17].

Micronutrient deficiencies are a major challenge in patients with NETs. As previously mentioned, vitamin B3 biosynthesis is impaired in carcinoid syndrome due to the common precursor with serotonin. Hypochlorhydria in gastric NETs, of autoimmune origin or upon treated Zollinger-Ellison syndrome, or in patients under SSA therapy, can lead to B12 deficiency, but B12 malabsorption can also be observed in patients with small intestine neoplasms or postoperatively. Vitamin D deficiency is also frequent in patients with NETs, particularly in those suffering from diarrhea or steatorrhea [9,15,17]. Possibly, this vitamin D deficiency is contributing to the increased prevalence of osteopenia and osteoporosis in these patients (41% and 10%, respectively), while higher levels of urinary 5-hydroxy-indoleacetic acid (5-HIAA), the metabolite of serotonin, could also play a role [32].

### 2.2. Treatment-Related Mechanisms

Beyond surgical and pharmacologic effects on digestion and absorption, accumulating evidence suggests that alterations of the gut microbiota may contribute to malnutrition and metabolic dysregulation in NETs. Patients with small bowel NETs appear particularly prone to small intestinal bacterial overgrowth (SIBO), driven by anatomical changes, impaired motility, use of proton pump inhibitors, and recurrent abdominal surgery, which may lead to bile acid deconjugation, carbohydrate malabsorption, steatorrhea, and micronutrient deficiencies. These microbiota-related mechanisms can amplify existing hormone-mediated diarrhea and malabsorption, thereby worsening weight loss and sarcopenia, and highlight the potential role of targeted diagnosis and management of SIBO (for example, with breath testing and evidence-based antibiotic or dietary strategies) as part of a comprehensive nutritional approach in selected NET patients [15].

Several different treatment modalities used in the therapeutic armamentarium of NETs can present as a side effect the aggravation of malnutrition and sarcopenia [33]. Previous surgeries in the gastroenteropancreatic tract can frequently cause alterations of the digestive tract, with subsequent reduced nutrition uptake. Gastrectomy or removal of the terminal ileum can lead to vitamin B12 deficiency, while small bowel resection can additionally cause bile acid malabsorption [34]. Furthermore, short bowel syndrome, but also bacterial overgrowth, can be present after both small intestine resection and the Whipple procedure. Whipple procedure, pylorus-preserving pancreatoduodenectomy, or distal pancreatectomy can also induce pancreatic exocrine insufficiency, causing steatorrhea and malabsorption of fat-soluble vitamins [9,22], but also impaired glucose metabolism [27].

Therapeutic administration of SSAs is often used to control gastrointestinal problems in patients with NETs [35]. SSA therapy can also be associated with abdominal pain, nausea, pancreatic exocrine insufficiency, and suboptimal nutritional absorption, and negative effects on nutritional status [27,36]. Moreover, due to their mechanism of action, SSAs may contribute to malnutrition and, consequently, to sarcopenia, highlighting the need for careful assessment and management of muscle mass and nutritional status in these patients.

Therapy with the mechanistic target of rapamycin (mTOR) inhibitor everolimus contributes to impaired glucose metabolism [37]. Furthermore, the mTOR pathway plays a pivotal role in activating skeletal muscle synthesis and the regulation of lipid and carbohydrate metabolism, with respective effects of its inhibitor on sarcopenia [38]. On the other hand, systemic chemotherapy can cause stomatitis, nausea, vomiting, diarrhea, all leading to malabsorption [9]. Peptide receptor radionuclide therapy can also cause fatigue, nausea and vomiting, but also pain and a carcinoid syndrome flare in the weeks following each cycle, together with diarrhea and, in particular, steatorrhea due to pancreatic insufficiency, leading to a transient reduction in food intake and subsequent weight loss [27,39]. Moreover, patients with small intestine NETs present an alteration of their gut microbiota, possibly affecting both malnutrition and carcinogenesis, rendering this a possible therapeutic target for the improvement of malnutrition, but also of prognosis [31].

### 2.3. Host-Related Factors

Survival in patients with NETs is significantly prolonged due to both better natural history of the disease per se, compared to classic cancer, but also due to new treatment modalities [40], so that the population with NETs is progressively aging. In this context, older adults with NETs are more susceptible to sarcopenia. In addition, the presence of comorbidities such as diabetes mellitus can aggravate this phenotype, whereas side effects of the various treatments mentioned before act additively and can complicate nutritional status [17,36].

## 3. Study Selection and Quality Appraisal

Given the narrative design and the heterogeneity of available data, a structured appraisal of methodological quality was undertaken for the main observational and interventional studies. Non-randomized observational cohorts examining malnutrition, sarcopenia, and outcomes in NETs were evaluated using domains adapted from ROBINS-I [41], focusing on confounding, selection of participants, classification and measurement of exposures/outcomes, missing data, and selective reporting. Across these studies, the overall risk of bias was generally judged as moderate to serious, primarily due to limited adjustment for key confounders (e.g., tumor burden, performance status, treatment line), single-center designs, and relatively small sample sizes, which may inflate or obscure associations between nutritional status, body composition, and clinical endpoints.

For interventional evidence (dietary, exercise, multimodal, and emerging pharmacologic or microbiome-targeted approaches), the certainty of evidence was appraised using a GRADE-informed approach at the level of each intervention category rather than per individual trial [42]. Most available data in NETs derive from small, often non-randomized or single-arm studies, frequently with short follow-up and reliance on surrogate outcomes such as changes in weight, skeletal muscle indices, or symptom scores, leading to an overall low to very low certainty of evidence for effects on hard outcomes, including survival, treatment tolerance, and long-term QoL. Consequently, while the direction of effect for early nutritional support and multimodal strategies is broadly consistent with findings in mixed-cancer cohorts, conclusions in NET-specific populations should be considered hypothesis-generating, and clinical recommendations must be interpreted with caution and individualized within multidisciplinary care.

## 4. Epidemiology and Clinical Significance

### 4.1. Prevalence of Nutrition-Related Problems

Malnutrition and sarcopenia are highly prevalent among patients with GEPNETs, with recent cohort studies using nutritional assessment and cross-sectional imaging reporting that nearly half of newly diagnosed or surgically treated patients meet the diagnostic criteria for sarcopenia or computed tomography (CT)-derived skeletal muscle indices [22,43]. With regard to GEPNENs, a substantial proportion of patients also fulfill the GLIM-malnutrition criteria, and malnutrition frequently coexists with sarcopenia and metabolic comorbidities such as diabetes, suggesting a complex interplay between tumor biology, hormonal syndromes, and host factors [17]. Although data directly applying GLIM and EWGSOP2 criteria for malnutrition and sarcopenia, respectively, in NET-specific populations remain limited, extrapolation from broader oncology cohorts indicates that these frameworks capture a clinically relevant burden of disease-related malnutrition that is likely underdiagnosed if only simple anthropometrics are used [44].

The co-existence of malnutrition and sarcopenia has important prognostic implications for patients with NETs. In patients with GEPNET, CT-defined sarcopenia has been associated with reduced overall survival and shorter progression-free survival, even after adjustment for conventional prognosticators [45]. More specifically, malnutrition roughly doubles mortality risk and increases postoperative complications [38], while sarcopenia identified using EWGSOP2 or imaging-based criteria is consistently linked to worse survival, higher treatment toxicity, and more frequent hospitalizations, patterns that are highly relevant to patients with NETs, given their long disease trajectories [46].

### 4.2. Nutritional Status, Prognosis, and Treatment Tolerance

Nutritional status and body composition have a significant impact on treatment tolerance and QoL in NETs. Based on an observational study in patients with advanced GEP-NENs, malnutrition and sarcopenia correlate with fatigue, gastrointestinal symptoms, and impaired health-related QoL scores, while symptom-directed therapies that improve diarrhea control can stabilize weight and reduce nutritional decline in patients with carcinoid syndrome [16,47]. According to the study of Ranallo et al., in NETs treated with targeted agents such as everolimus, baseline low muscle mass and the phenotype of sarcopenia have been associated with higher rates of dose reductions or interruptions, underlining the potential role of sarcopenia as a predictor of treatment-related toxicity [38]. These findings underpin the importance of early detection of nutrition-related problems and functionality deterioration, to treat early and sufficiently therapy-limiting complications over the course of chronic NET management [19].

An additional consideration is the temporal relationship between the onset of malnutrition or sarcopenia and the initiation of systemic therapy in NETs. Baseline malnutrition and low skeletal muscle mass at the time of diagnosis or before treatment have been associated in broader oncologic cohorts with higher postoperative morbidity, increased treatment toxicity, and poorer survival, suggesting that nutritional risk present at the outset may confer a different prognostic profile than deterioration that develops later under therapy [15]. In NET populations, observational data similarly indicate that patients often enter PRRT, targeted therapies, or cytotoxic regimens with pre-existing weight loss, muscle depletion, and micronutrient deficiencies, which may already compromise treatment tolerance and functional reserve. This underscores the need to embed systematic nutrition and body composition assessment into the pre-treatment work-up and to distinguish early, potentially modifiable deficits from end-stage cachectic trajectories when interpreting outcomes and designing interventions [16,17].

## 5. Assessment and Diagnosis

### 5.1. Nutritional Screening—Assessment

Nutritional screening is the first step for identifying patients at risk of malnutrition. Validated nutritional screening tools such as the NRS-2002, the MUST, and the PG-SGA are widely recommended in oncology and have been shown in mixed-cancer cohorts to correlate with GLIM-diagnostic criteria of malnutrition. More specifically, NRS-2002 is demonstrating particularly good agreement, and PG-SGA offers high sensitivity for detecting malnutrition [10,44]. In clinical practice, these tools can be used at baseline and at regular intervals to early identify patients who would require more detailed nutritional assessment, including the evaluation of dietary intake, the nutrition-related symptoms, changes in body weight, and in functional status [21].

### 5.2. Body Composition Analysis

Objective assessment of body composition is pivotal for the diagnosis of sarcopenia and the quantitative and qualitative estimation of body mass changes in patients with NETs [39]. The evaluation of analysis of muscle mass based on the analysis of images of CT, at the third lumbar vertebra on routine staging or restaging scans, is considered a reference method for quantifying muscle mass and identifying sarcopenia in oncology [48,49], and has been successfully applied in patients with NET to demonstrate high prevalence muscle mass deterioration and to explore associations with survival and treatment outcomes, with reduced overall survival (OS)/progression-free survival (PFS) (Hazard Ratio, HR) 1.8–2.5 across cohorts) [22,34,45]. Magnetic resonance imaging (MRI) offers similar capabilities with superior soft-tissue contrast and radiation exposure, but it is less standardized for body composition analysis; dual-energy X-ray absorptiometry (DXA) and bioelectrical impedance analysis (BIA) are usually more accessible in a clinical setting, but the estimation of lean body mass and fat mass may be affected by the fluid status of the patients. Apart from that, based on the study by Kroll et al., body composition analysis based on Computed Tomography (CT) scans can significantly reduce the cost as regular staging exams can be used for the analysis, and at the same time limit the radiation exposure from DXA, providing a significant improvement of QoL in patients with GEPNET [50].

In the available studies on NET populations, sarcopenia was predominantly quantified using cross-sectional imaging, yet substantial methodological heterogeneity exists in how skeletal muscle was assessed and classified. Investigators have applied different vertebral landmarks (most commonly the third lumbar vertebra, but in some cohorts the third cervical or other levels), variable segmentation protocols, and a range of cut-off values for skeletal muscle index or muscle attenuation, while some reports relied solely on muscle quantity without incorporating strength or performance measures as recommended by EWGSOP2 [13]. This variability complicates direct comparison of sarcopenia prevalence and effect estimates across studies and may dilute the apparent strength of associations between sarcopenia, treatment tolerance, and survival; therefore, the prognostic inferences presented in this review should be interpreted as hypothesis-generating and viewed in the context of these methodological differences. Harmonization of CT-based protocols and incorporation of functional testing in future NET-specific research will be crucial to refine risk stratification and to support more robust, generalizable conclusions. Table 1 summarizes the nutritional screening tools, the assessment tools, and the diagnostic criteria commonly used in populations with NETs.

### 5.3. Biomarkers

Biomarkers can be used as a complementary measure for clinical and imaging assessment, reflecting systemic inflammation, visceral protein status, and anabolic-catabolic balance, all of which influence nutritional and muscle health. Serum albumin, although affected by factors beyond nutrition, remains a widely used marker associated with prognosis and perioperative risk, while C-reactive protein (CRP) and composite indices incorporating CRP and albumin are used to evaluate the inflammatory component of cancer cachexia. Apart from that, CRP is used as an aetiologic criterion for malnutrition diagnosis based on GLIM criteria [10,51].

Within the GLIM framework, biomarkers such as serum albumin and CRP are often considered in the context of inflammation and disease burden, yet their interpretation in NET populations requires caution. Albumin is a negative acute-phase reactant strongly influenced by systemic inflammation, hepatic function, fluid status, and overall disease severity, and therefore, low concentrations may reflect catabolic and inflammatory activity rather than isolated nutritional depletion, especially in patients with advanced tumors or treatment-related toxicity [20]. Similarly, CRP is endorsed as an etiologic indicator of inflammation in GLIM, but in NETs, it may be chronically elevated due to tumor-related inflammatory pathways, infections, or therapy complications, complicating its attribution to malnutrition per se and potentially leading to overestimation of the inflammatory component of GLIM-defined malnutrition [10,51]. In this context, biomarker changes should be integrated with clinical assessment, body composition measures, and functional outcomes, and future NET-specific studies are needed to validate GLIM-based biomarker thresholds against hard endpoints such as survival, treatment tolerance, and quality of life to refine their use in this setting.

Additional markers such as insulin-like growth factor 1 (IGF-1), vitamin D, and muscle-derived cytokines and myokines have been investigated as potential indicators of muscle mass and function in patients with cancer, but their roles in NET-specific settings are not yet firmly established and are primarily used in research rather than routine care [52,53].

## 6. Nutritional and Metabolic Interventions

Interventions aiming to improve nutritional status and body composition in patients with NETs require a multidisciplinary approach, addressing changes in dietary intake and behavior, physical activity, artificial nutritional support, and pharmacological agents. This multimodal strategy focuses on the management of malnutrition and sarcopenia, thereby improving QoL and clinical outcomes [54].

In clinical practice, the timing of nutritional and metabolic interventions appears crucial. Evidence from mixed-cancer populations suggests that supportive care initiated before or at the very beginning of systemic therapy is more likely to stabilize weight, maintain function, and reduce dose-limiting toxicities than interventions introduced only at advanced or refractory stages, when catabolic drive and symptom burden are pronounced [17]. For patients with NETs, this supports a strategy of proactive, early screening and individualized intervention planning around key treatment milestones—such as planned surgery, PRRT, or commencement of targeted agents—rather than a reactive approach triggered only by severe weight loss or overt functional decline [15]. To facilitate the early intervention, nutritional screening should be implemented at diagnosis or before the initiation of major treatments (surgery/ PRRT) and repeated every 3 months during active therapy or every 6 months in stable follow-up [17,21,55]

### 6.1. Dietary Interventions

Several factors, namely the tumor-induced metabolic alterations and malabsorption, change energy and nutrient needs in patients with NET. As there are no specific guidelines for patients with NETs regarding the recommended energy and protein intake, the guidelines of the European Society of Clinical Nutrition and Metabolism for patients with cancer can be used [21,55]. Energy targets should be set at 25–30 kcal/kg/day, while protein intake should be increased to reach 1.2–1.5 g/kg/day (or 1.5–2.0 g/kg in sarcopenia/severe malnutrition), adjusted for diarrhea/renal function [21,55].

Dietary modification plays a key role in symptom control for patients with carcinoid syndrome, though evidence remains largely observational [24,25]. Common triggers include high histamine/tyramine foods and specific beverages that may precipitate flushing, diarrhea, or abdominal discomfort via biogenic amine release or vasoactive effects [27]. Rather than universal avoidance—which increases the risk of malnutrition—current expert guidance recommends individualized testing via 1–2-week food-symptom diaries followed by selective trigger reduction under dietitian supervision, ensuring maintenance of energy targets. In Table 2, the common nutritional triggers for carcinoid syndrome and dietary alternatives are presented.

Dietary counseling should be provided on an individual basis to identify and treat nutrition-related symptoms that compromise nutritional status early in the course of the disease. Nutrition modification may involve the number of meals, adjustments in fat and fiber intake, and increases in energy and protein, to facilitate improved tolerance and the preservation of body weight and muscle reserves [15,21,55]. Pancreatic enzyme replacement therapy (PERT) can effectively manage exocrine pancreatic insufficiency, which is common in patients with NETs or postoperatively in patients who have undergone pancreatic surgery. PERT improves fat absorption by reducing steatorrhea and helps in the restoration of nutritional status and muscle mass. To achieve these results, the management of PERT doses should be based not solely on the improvement of gastrointestinal symptoms but on the achievement of nutritional intake goals [56].

For patients not covering their nutritional needs through their diet, food fortification and oral nutritional supplements (ONS) can be considered, especially during treatments [55,57]. For the ONS studies in patients with cancer have shown that high protein supplements containing omega-3 fatty acids can improve nutritional and inflammatory markers, resulting in shorter length of stay and overall improved clinical outcomes [58]. Importantly, the evidence base for ONS in NETs is almost entirely extrapolated from broader oncology cohorts, as NET-specific ONS trials are currently lacking. Recent meta-analyses and systematic reviews in mixed-cancer settings indicate that high-protein, energy-dense ONS can improve or stabilize body weight and, in some studies, selected QoL domains, particularly in patients at nutritional risk or undergoing active treatment. However, these benefits are not uniform across all outcome measures: changes in serum albumin, CRP, and other inflammatory or visceral protein biomarkers are often modest or inconsistent, and improvements in comprehensive tools such as the PG-SGA may vary by population and study design. Consequently, ONS in NETs should be viewed as a pragmatic component of a broader, individualized nutrition strategy aimed at supporting intake, symptom control, and functional status, rather than as a stand-alone intervention expected to normalize laboratory markers in isolation.

Enteral nutrition can also be considered in advanced disease stages and severe malnutrition. It can cover the needs of patients, not tolerating oral nutrition. For those with severe malabsorption or bowel dysfunction, parenteral nutrition, supplemental or total parenteral nutrition, can also be provided [55]. More specifically, in patients failing to achieve <60% requirements despite counseling/ONS for >7–10 days, enteral nutrition support should be considered, if gut function). If enteral nutrition is contraindicated or not tolerated or is covering less than 50% of the patient’s needs, supplemental or total parenteral nutrition should be considered, especially in patients with short bowel or severe steatorrhea [17,55]

Special care should be taken for patients with malabsorption and hormone hypersecretion. In these patients, vitamin and trace element deficiencies are common, especially B complex, vitamin D, and the other fat-soluble vitamins [9].

### 6.2. Exercise and Physical Activity

As the main therapeutic aim is the preservation or improvement of muscle mass and function, resistance and combined training are generally recommended, with evidence in broader oncology and geriatric sarcopenia populations showing that such programs can stimulate muscle protein synthesis, enhance mitochondrial function, and improve physical performance [59]. Nevertheless, the strength of evidence supporting exercise interventions specifically in patients with NETs remains limited. Existing data largely derive from small randomized or quasi-experimental trials and observational studies in gastrointestinal or endocrine cancers that include but do not focus exclusively on NETs, as well as expert consensus statements such as the recent ENETS position paper on nutritional support [17]. To date, randomized controlled trials designed explicitly for NET cohorts and powered to evaluate functional outcomes, body composition, and treatment tolerance are sparse, so current recommendations for structured, personalized exercise in NETs should be regarded as extrapolated from related populations and prioritized as a key area for future research [60].

The ENETS position statement on nutritional support in neuroendocrine neoplasms explicitly recommends integrating structured physical activity and resistance exercise into multimodal care for NET patients, with tailoring to symptom burden, comorbidities, and treatment phase [17]. In addition, NET nutrition resources and expert guidance advise incorporating regular walking and simple resistance exercises to counteract sarcopenia and treatment-related fatigue, while emphasizing the need to individualize session duration and intensity in those with carcinoid-related diarrhea, PRRT-related fatigue, or advanced disease [61].

### 6.3. Multimodal Interventions

**Figure 2:**
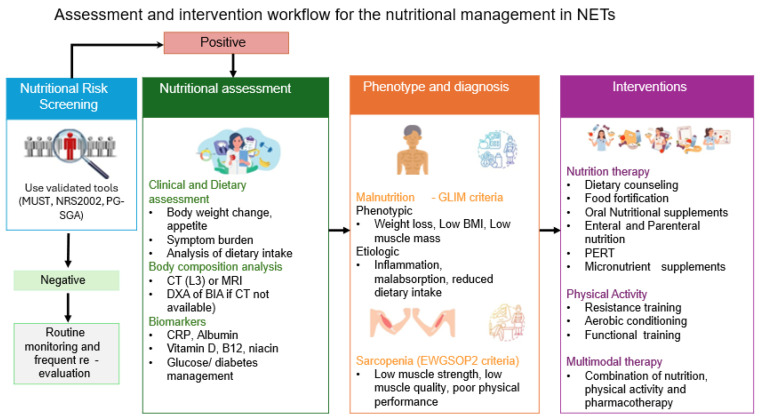
Nutritional screening, assessment, and management of patients with NETs. Footnote: NRS2002: Nutritional Risk Screening 2002, MUST: Malnutrition Universal Screening Tool, CT: Computed tomography, BIA: Bioelectrical Impedance Analysis, DXA: Dual-energy X-ray Absorptiometry, BMI: Body mass index, NETs: Neuroendocrine tumors, CRP: C-reactive protein, PERT: Pancreatic Enzyme Replacement Therapy, GLIM: Global Leadership Initiative on Malnutrition, EWGSOP2: European Working Group on Sarcopenia in Older People 2, L3: 3rd lumbar disc.

Multimodal interventions combine nutrition, exercise, and anti-inflammatory treatment to treat cancer cachexia and sarcopenia efficiently. This type of intervention has been described in mixed cancer Randomized Controlled Studies (RCTs), such as the Multimodal-Exercise, Nutrition and Anti-inflammatory medication for Cachexia (MENAC) trial. Based on the rationale of this trial, the effects of the combination of interventions aim to improve muscle mass, functionality, nutritional status, and treatment tolerance [62]. It should be stressed, though that studies on patients with NETs remain limited. Therefore, the application of this type of intervention in patients with NETs should be adopted to tumor-specific factors, including the chronicity of symptoms and the hormonal syndromes, and should be applied by a multidisciplinary team of oncologists, endocrinologists, surgeons, gastroenterologists, nutritionists, nurses, psychologists, physiotherapists, and exercise specialists [17]. The steps of nutrition screening, assessment, and intervention are summarized in Figure 2.

## 7. Emerging and Experimental Interventions

Even if nutritional intervention is the cornerstone of malnutrition and sarcopenia treatment, nowadays, new therapeutic approaches emerge. For the purposes of this narrative review, “emerging interventions” are defined as novel or not yet routinely implemented strategies that specifically target malnutrition, sarcopenia, or their underlying metabolic and inflammatory pathways in NETs. These approaches go beyond standard nutritional counseling, symptom-directed pharmacotherapy and conventional exercise prescriptions, and include microbiome-modulating strategies (such as probiotics, prebiotics, or dietary patterns designed to favorably alter the gut–muscle axis), orexigenic and anabolic agents (e.g., ghrelin receptor agonists and myostatin-pathway inhibitors), as well as digital health and artificial-intelligence–enabled tools that individualize dietary, activity and body-composition monitoring. Given the very limited number of NET-specific trials, most of these interventions are currently supported by early-phase data or extrapolation from broader oncology and geriatric populations and should therefore be considered exploratory.

Among emerging and experimental interventions, modulation of the gut microbiota represents a particularly promising but still exploratory field [63]. The gut–muscle axis, mediated in part by microbial metabolites such as short-chain fatty acids, has been implicated in the regulation of energy balance and muscle homeostasis, and dysbiosis has been described in patients with small bowel NETs, especially those with SIBO and bile acid malabsorption [15,22]. Pilot data from non-NET cancer and geriatric populations suggest that targeted dietary patterns, probiotics, prebiotics, and, in selected cases, antibiotics can influence symptoms, inflammatory markers, and body composition [64,65], but high-quality NET-specific trials are lacking [15,17]. Accordingly, microbiome-directed strategies in NET-related malnutrition and sarcopenia should currently be considered investigational adjuncts within multidisciplinary care, ideally implemented in the context of clinical studies.

The gut microbes-muscle axis is an important regulator of muscle homeostasis, and particularly microbial metabolites like short-chain fatty acids, such as acetate, propionate, and butyrate, exert effects on host energy balance [66]. Different exercise modalities (endurance, moderate or high intensity, resistance, and aerobics) have respective effects on microbiome and thereby can act as therapeutic modulators of the gut-muscle axis, combating muscle wasting in sarcopenia [66]. As previously mentioned, patients with NETs display altered ghrelin expression. Ghrelin receptor agonists have been shown to stimulate appetite, improve body composition, and reverse muscle mass decline [67]. Similarly, myostatin inhibitors have shown promising effects in enhancing strength in preclinical mouse models, and although clinical studies have not substantiated these observations so far, these compounds could possibly serve as a therapeutic option for a range of muscle-wasting disorders [68,69]. Finally, the application of digital technologies such as machine learning and artificial intelligence in nutrition-related diseases, such as diabetes, obesity, but also sarcopenia, could be applied for the management of patients, and also predict outcomes based on a personalized approach [70,71].

Overall, the current evidence base for emerging interventions in NET-related malnutrition and sarcopenia remains sparse and heterogeneous, with small sample sizes, short follow-up, and frequent reliance on surrogate endpoints such as changes in body composition rather than hard clinical outcomes. When appraised using structured certainty-of-evidence frameworks, most data on microbiome modulation, ghrelin agonists, myostatin inhibition, and digital/AI-supported interventions would be classified as low to very low certainty, underscoring the need for adequately powered, NET-focused trials with standardized nutritional and functional endpoints. Until such data becomes available, these strategies should be implemented cautiously, ideally within research protocols or specialized multidisciplinary programs, and always in conjunction with established nutritional and exercise interventions rather than as stand-alone therapies.

## 8. Conclusions

Malnutrition and sarcopenia are very common in patients with NETs. The complex interplay of tumor biology, the hormone-mediated metabolic alterations, the chronic treatment effects, and the symptom burden has a negative impact on nutritional intake, increasing malabsorption and overall metabolic needs. Despite the limited data on patients with NETs, emerging evidence supports the need for a multidisciplinary approach incorporating individualized nutritional support, pharmacological therapy, exercise-based rehabilitation, and integrated multimodal care. Early identification of malnutrition risk and sarcopenia through validated screening and body composition assessment tools is paramount to optimize outcomes, preserve physical function, and improve QoL. Future research should focus on tailored interventions that address the unique pathophysiological and clinical features of patients with NETs. Multidisciplinary collaboration among oncologists, endocrinologists, nutritionists, nurses, physiotherapists, and exercise specialists is essential to provide personalized, evidence-based nutritional and metabolic care for these patients.

## Figures and Tables

**Table 1:** Nutritional screening and assessment tools and diagnostic criteria for malnutrition and Sarcopenia in NETs.

Tool/Framework	Type	Primary Scope	Key Components	Main Strength	Key Limitation	Use in NETs/Oncology
NRS2002	Screening tool	Nutritional risk screening	Recent weight loss, BMI, reduced intake, disease severity, and age adjustment	Simple, quick, validated, hospitalized, and oncology patients, good agreement with GLIM	Does not directly assess body composition or sarcopenia; less sensitive to micronutrient deficiencies	Commonly used at diagnosis and during treatment in cancer and NET cohorts to identify patients at risk of malnutrition, who need nutritional assessment
MUST	Screening tool	Nutritional risk screening	BMI, unintentional weight loss, and acute disease effect	Simple, quick, and easy to apply in outpatient/ clinic settings; widely used in cancer	May underestimate risk in patients with fluid retention or preserved BMI, but muscle loss	Applied in some NET studies and general oncology clinics, especially in ambulatory settings
PG-SGA	Assessment tool	Comprehensive nutritional assessment	Symptoms, dietary intake, weight change, functional impact, physical exam	Oncology specific; high sensitivity for malnutrition; guides targeted intervention	Requires more time and trained staff	Frequently used as a reference for malnutrition diagnosis and for monitoring response to nutrition support in cancer and selected NET cohorts
GLIM criteria	Diagnostic criteria	Diagnostic framework for malnutrition	Phenotypic (weight loss, low BMI, reduced muscle mass) plus etiologic (reduced intake, inflammation/disease burden) criteria	International consensus; links screening to diagnosis; adaptable across settings	Requires body composition or surrogate; interpretation of inflammation markers (CRP, albumin) can be challenging in cancer	Increasingly applied in oncology and beginning to be used in NET studies to quantify malnutrition burden.
EWGSOP2 criteria	Diagnostic criteria	Diagnostic framework for sarcopenia	Muscle strength (grip, chair stand), muscle quantity/quality (DXA, CT, BIA, MRI), physical performance (gait speed, SPPB)	Emphasizes function plus mass; clear staging (probable, confirmed, severe)	Requires equipment and time; not routinely implemented in oncology clinics	Used as a conceptual reference in studies for NET, the full EWGSOP2 application remains rare in NET cohorts due to resource constraints.
CT derived skeletal muscle index	Body composition analysis	Quantification of muscle mass/ imaging-defined sarcopenia	Cross-sectional muscle area at the lumbar (often L3) or cervical (C3) level, normalized for height	Uses routine staging CT; highly reproducible; strong prognostic value in many cancers	No direct functional measure; heterogeneous cut-offs and vertebral levels; influenced by edema	Widely used in GEP-NET studies to estimate sarcopenia prevalence and associations with survival and treatment toxicity, though methods and thresholds vary
BIA	Body composition analysis	Bedside body composition assessment	Whole-body or segmental electrical conductivity to estimate fat-free mass, fat mass, phase angle, and extracellular water	Portable, non-invasive, rapid (<5 min), relatively inexpensive; good for serial monitoring and hydration status	Highly sensitive to hydration/fluid shifts (common in cancer/NETs with diarrhea/edema); requires standardized conditions; less accurate in extreme BMI	Used in NET research for tracking treatment effects on lean mass and phase angle; valuable for ambulatory monitoring, but needs cautious interpretation
DXA	Body composition analysis	Precise body composition and bone assessment	Whole-body or regional scanning for bone mineral density, lean soft tissue mass, and fat mass	Gold standard for body composition; precise ASMM; simultaneous bone assessment	Requires specialized equipment; radiation exposure (low); not bedside; limited availability in oncology settings	Applied in select NET studies for accurate sarcopenia phenotyping and osteoporosis assessment; ideal research tool, but rarely routine in NET clinics.

NRS2002: Nutritional Risk Screening 2002, MUST: Malnutrition Universal Screening Tool, GLIM: Global Leadership Initiative on Malnutrition, EWGSOP2: European Working Group on Sarcopenia in Older People 2, CT: Computed tomography, BIA: Bioelectrical Impedance Analysis, DXA: Dual-energy X-ray Absorptiometry, MRI: Magnetic resonance imaging, SPPB: Short Physical Performance Battery, ASMM: appendicular skeletal muscle mass, BMI: Body mass index, NETs: Neuroendocrine tumors, CRP: C-reactive protein

**Table 2:** Common nutritional triggers for carcinoid syndrome.

Trigger Category	Foods to Limit/Avoid	Alternatives
High-histamine/tyramine food items	Aged cheeses (cheddar, blue), cured meats (salami, bacon), fermented foods (sauerkraut, soy sauce)	Fresh mozzarella/cottage cheese, fresh poultry/fish, homemade broths
Beverages	Alcohol (red wine, beer), caffeine	Herbal teas, diluted fruit juices, and water with lemon
Other triggers	Chocolate, spicy foods, walnuts, yeast extracts	Carob/white chocolate, mild herbs, pumpkin seeds, fresh bread

## Data Availability

No new data were created or analyzed in this study.

## Abbreviations

The following abbreviations are used in this manuscript:

NENs	Neuroendocrine neoplasms
GEP	Gastroenteropancreatic
BP	Bronchopulmonary
NETs	Neuroendocrine tumors
NECs	Neuroendocrine carcinomas
QoL	Quality of life
PRRT	Peptide receptor radionuclide therapy
BMI	Body mass index
GHSR1a	Growth hormone secretagogue receptor 1-a
GHSR1b	Growth hormone secretagogue receptor 1-b
GI	Gastrointestinal
SIBO	Small intestine bacterial overgrowth
PEI	Pancreatic exocrine insufficiency
5-HIAA	5-hydroxy-indoleacetic acid
SSAs	Somatostatin analogs
mTOR	Mechanistic target of rapamycin
CT	Computed tomography
GLIM	Global leadership initiative on malnutrition
EWGSOP2	European Working Group on Sarcopenia in Older People 2
NRS-2002	Nutritional Risk Screening 2002
MUST	Malnutrition Universal Screening Tool
PG-SGA	Patient-Generated Subjective Global Assessment
MRI	Magnetic resonance imaging
DEXA	Dual-energy X-ray absorptiometry
BIA	Bioelectrical impedance analysis
CRP	C-reactive protein
IGF-1	Insulin-like growth factor 1
PERT	Pancreatic enzyme replacement therapy
ONS	Oral nutritional supplements
MENAC	Multimodal-Exercise, Nutrition and Anti-inflammatory Medication for Cachexia
